# Enhanced study of facial soft tissues using a novel large scale histology technique

**DOI:** 10.1002/ca.23943

**Published:** 2022-08-17

**Authors:** Lennert Minelli, Rory George Charles Bown, Erica Wung Hwa Mu, Darryl Lane Whitehead, Tania Helen Henderson, Felicity Lawrence, Ian Mellor, Matthew Ian Wissemann, Cameron Peter Brown, Berend van der Lei, Bryan Christopher Mendelson

**Affiliations:** ^1^ Melbourne Advanced Facial Anatomy Course (MAFAC) Australasian Society of Aesthetic Plastic Surgeons Toorak Australia; ^2^ Medical Engineering Research Facility Queensland University of Technology Brisbane Australia; ^3^ Department of Plastic Surgery, University Medical Centre Groningen University of Groningen Groningen The Netherlands; ^4^ School of Biomedical Sciences The University of Queensland Saint Lucia Australia; ^5^ Central Analytical Research Facility Queensland University of Technology Brisbane Australia

**Keywords:** aesthetic surgery, anatomy, connective tissue, cosmetic surgery, facelift, facial nerve, fascia, histology, rhytidectomy

## Abstract

The safety and effectiveness of facial cosmetic surgery procedures are dependent on detailed 3D understanding of the complex surgical anatomy of the face. Traditional, small sample size anatomical dissection studies have limitations in providing definitive clarification of the fascial layers of the face, and especially in their relationship with the facial nerve, and their reaction to surgical manipulation. The objective study of large tissue areas is required to effectively demonstrate the broader architecture. Conventional histology techniques were modified to handle extraordinarily large tissue samples to fulfill this requirement. Full‐thickness soft tissue samples (skin to bone) of maximum length 18 cm, width 4 cm, and tissue thickness 1 cm, were harvested from 20 hemifaces of 15 fresh human cadavers (mean age at death = 81 years). After fixation, the samples were processed with an automated processor using paraffin wax for 156 h, sectioned at 30 μm, collected on gelatin‐chromium‐coated glass slides, stained with a Masson's Trichrome technique and photographed. Using this technique, excellent visualization was obtained of the fascial connective tissue and its relationship with the facial mimetic muscles, muscles of mastication and salivary glands in 73 large histological slides. The resulting slides improved the study of the platysma and superficial musculo‐aponeurotic system (SMAS), the spaces and ligaments, the malar fat pad, and the facial nerve in relations to the deep fascia. Additionally, surgically induced changes in the soft‐tissue organization were successfully visualized. This technique enables improved insight into the broad structural architecture and histomorphology of large‐scale facial tissues.

## 1 INTRODUCTION

Understanding the structural architecture of the facial soft tissues is fundamental for safe and effective facial surgery, including elective procedures involving appearance; this includes knowledge of the architecture of fascial layers with their adaptions for movement and their relationship with the facial nerve. In aesthetic surgery of the face, in addition to this core surgical knowledge, understanding of the changes of the anatomy with aging and the response of the tissues to surgical manipulation is required, with the focus on obtaining natural appearing outcomes with positive long‐term results with minimization of recurrent tissue ptosis.

The lack of a complete anatomical overview with details of the facial soft tissue architecture may contribute to the shortcoming of current surgical techniques (Hamra, 2002). Moreover, adoption of these techniques by inexperienced surgeons is hindered by the absence of clear and unambiguous descriptions of the anatomy pertaining to the layers and commonly used dissection planes. Knowledge of anatomical structural features, such as tissue layers, gliding planes, ligaments, spaces, fusion zones, and so forth in the face still remains largely based on traditional anatomical dissection and surgical experience, occasionally leading to conflicting descriptions (Mendelson & Wong, 2016, 2018; Pessa, 2016; Zins & Hashem, 2016).

Objective technical investigations, such as histology, computed tomography (CT) and magnetic resonance imaging (MRI), are required to complement dissection findings, but have their limitations, which reduce their value in the study of facial soft tissues. Both CT and MRI lack the spatial resolution for the required microscopic visualization of the facial nerve branches in relation to the different fascial sheets. Histologic investigations, while ideal to demonstrate the structural architecture of soft tissues, are restricted by sample size. Standard histology techniques focus on small biopsy sizes 1–2 cm (L/W) × 0.5 cm (D) to allow optimal fixation and processing during tissue preparation. Traditional methods for embedding and sectioning of larger specimens (e.g., in nitrocellulose) are hazardous, time‐consuming, and increasingly expensive (Gray, 1954; Mann, 1902). More recently described methods to study larger samples of soft tissues still fall short of the necessary size needed to study the complex soft tissue architecture of the face (Bryant et al., 2019).

To overcome this limitation, numerous adjustments were made to our routine histological methods to enable production of large‐scale samples using conventional and affordable materials, allowing more labs to use this technique for their research. The goal of this publication is to provide the detailed methodology and histological technique to encourage clinical anatomists to investigate and report on large facial samples, of up to L 18 × W 4 × D 1 cm.

## 2 MATERIALS AND METHODS

Ethical approval for the project was granted by the University Human Research Ethics Committee of the Queensland University of Technology (Project number LR 2021‐4306‐4761). The authors state that every effort was made to follow all local and international ethical guidelines and laws that pertain to the use of human cadaveric donors in anatomical research. From September 2021 to February 2022, 20 hemifaces of 15 human cadavers were investigated (*n* = 15; male = 9; female = 6; mean age at death = 81 years). Two fresh‐frozen bodies were used for methodology optimization and nine fresh non‐frozen bodies, one embalmed body and three fresh‐frozen bodies were used to produce histological slides using the described histological method. Five cadavers underwent a surgical intervention to the unilateral face prior to harvesting to allow comparison with the unaltered side.

### 2.1 Histological technique

#### 2.1.1 Harvesting and preserving

The hemiface is dissected, sutured to firm cardboard, and then submerged in formalin (10% Neutral Buffered Formalin) at a 10:1 volume ratio of chemical fixative to facial tissue. Following 5 days of fixation, manual sub‐sectioning of each hemiface into equally long but thinner samples is performed using a brain knife. The resulting samples, L 5–18 × W 1–4 × D 0.7–1 cm, are individually sutured flat to cardboard at four corners and resubmerged in formalin for another 48 h (Video S1).

#### 2.1.2 Processing

Fixed tissue samples are washed in slow running distilled water for 2 h at room temperature to remove excess fixative remaining from storage in the formalin solution. Afterwards, tissue samples are placed into 30% ethanol, followed by 50% ethanol for 2 h each at room temperature.

The tissue samples are loaded into processing trays and processed through a Leica ASP300S Pathcentre. A 6‐day automated cycle is set with processing times detailed in Table 1. Samples are processed through ascending concentrations of ethanol, cleared in xylene, and infiltrated with molten paraffin wax for 24 h in each solution, while under a vacuum of −70 kPa and impregnation pressure of 35 kPa. All steps of the process occur at 35°C, except for the wax stations, which are at 65°C. Subsequently, the wax infiltrated samples are stored in zip‐lock bags at 4°C.

**TABLE 1:** Schedule for tissue processing of the large tissue samples

Reagent	Typical duration for regular histology samples (h)	Duration for current large samples (h)
Ethanol 70%	0:45	16:00
Ethanol 90%	0:45	12:00
Ethanol 95%	0:20	06:00
Ethanol 95%	0:20	06:00
Ethanol 100%	0:30	12:00
Ethanol 100%	0:30	12:00
Ethanol 100%	0:30	12:00
Xylene	0:30	12:00
Xylene	0:30	12:00
Xylene	0:30	12:00
Paraffin wax	0:30	12:00
Paraffin wax	0:30	12:00
Paraffin wax	1:00	24:00

#### 2.1.3 Embedding

**FIGURE 1:**
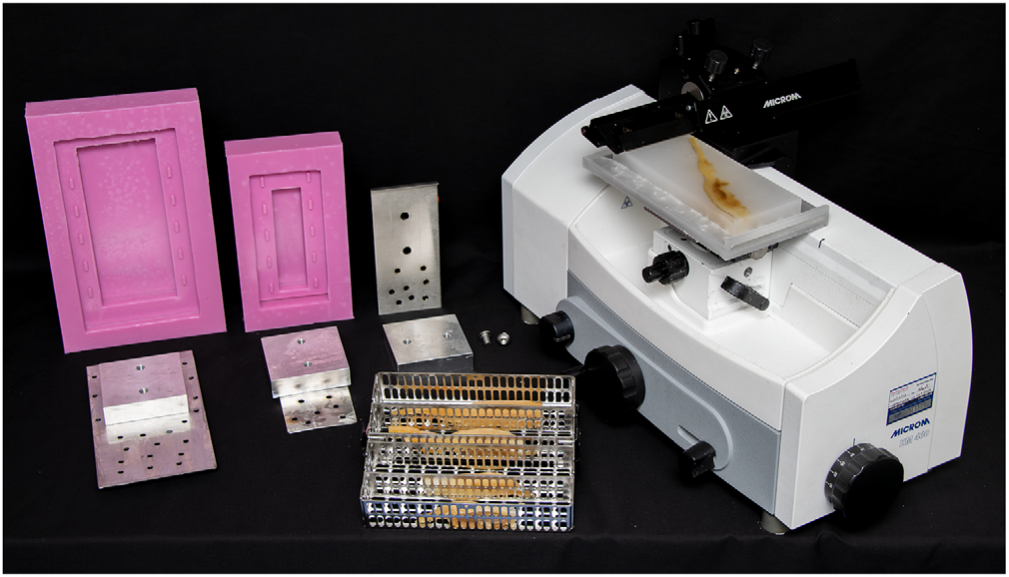
Equipment used for this technique. From left to right: Custom made silicon molds and mounting stages composed of a plate and a base, Pathcentre trays with tissues, sliding microtome. Aluminum mounting plates were made in two sizes to accommodate the varying tissue lengths, a 10 × 20 cm plate for larger specimens and a 6.5 × 15 cm plate for smaller specimens. The processor, Leica ASP300S Pathcentre, is not shown in this image

3D‐printed soft silicone molds (Pinkysil® RTV Silicone Rubber, Barnes, AU) and custom‐made aluminum mounting stages are used (Figure 1). The mounting stages feature two parts: a mounting plate of 2–4 mm thick aluminum sheet metal with 5 mm perforations to allow wax penetration and air bubble release, and a mounting base of 7.5 × 7.5 × 2 cm square aluminum metal that can be screwed onto the mounting plates prior to embedding. Each sample is embedded to obtain a longitudinal sectioning orientation parallel and close to the surface of the wax block face. Molten paraffin wax is poured slowly and incrementally into the mold allowing the lower portion of the mold to partially thicken while the sample is held in place with forceps. The mounting stage is positioned on top and, together, they are carefully transferred onto a cooling plate. Once the wax has set and is firmly attached to the mounting stage, the silicone mold is removed, and the sample block is stored at 4°C prior to sectioning (Video S2).

#### 2.1.4 Sectioning and staining

Embedded blocks are placed into the stage lock clamp of a Microm HM 430 sliding microtome. The blade holder is positioned at a 5° angle, and a disposable blade is inserted (Epredia, Bio‐Strategies, Aus). Sections are cut at 30 μm, gathered with the use of forceps and a paintbrush, and floated onto a warm gelatinized water bath at 42°C containing thymol preservative (Video S3).

Sections are collected onto large size gelatin‐chromium‐coated glass slides (12.5 × 17.5 cm). Coating of these plain glass slides is done prior, by dipping in a gelatin‐chromium adhesive solution (1.25% gelatin and 0.125% chromium potassium sulfate in distilled water) and allowing to dry at room temperature for a minimum of 24 h.

Specimen slides are dried overnight at room temperature and then baked in a 37°C oven for 30 min prior to staining. The sections are stained with a Masson's Trichrome stain, summarized in Table 2. Coverslips are mounted with a non‐aqueous hard setting mounting medium (DPX Neutral Mounting Medium). The slides are placed into a 37°C oven to harden prior to imaging.

**TABLE 2:** Modified Masson's Trichrome staining technique for large histological samples^a^

Main steps	Step‐by‐step actions
Dewaxing	Xylene (1 min)
	Xylene (2 min)
	Xylene (2 min)
Rehydrating	Ethanol 100% (2 min)
	Ethanol 100% (3 min)
	Ethanol 90% (2 min)
	Ethanol 70% (2 min)
	Distilled running water (2 min)
Staining	Weigert's Iron Hematoxylin (8 min)
	Distilled running water (5 min)
Staining	Biebrich scarlet‐acid fuchsin solution (12 min)
	Distilled running water (5 min)
Differentiation	Phosphomolybdic‐phosphotungstic acid solution (5 min)
	Phosphomolybdic‐phosphotungstic acid solution (10 min)
Staining	Acidic aniline blue solution (7 min, directly from the phosphomolybdic‐phosphotungstic acid solution without rinse)
	Distilled running water (5 min)
Differentiation	1% glacial acetic acid for 10 min
	Distilled running water (5 min)
Rapid dehydrating	Ethanol 95% (10 dips)
	Ethanol 100% (20 dips)
	Ethanol 100% (20 dips)
	Ethanol 100% (20 dips)
Clearing	Xylene (2 min)
	Xylene (2 min)
	Xylene (2 min)

^a^ See supplementary material for detailed recipe and instructions for making the solutions.

#### 2.1.5 Imaging

Excess mounting medium on the slides is removed with a straight edge blade and slides are cleaned with ethanol. The slides are imaged with a fixed zoom and settings regardless of the tissue size (Nikon D780 camera with an AF‐S micro NIKKOR 105 mm 1:2.8G ED lens), obtaining approximately ¼ of the tissue size per image. Numerous images of each sample were white‐balanced and merged in Adobe Photoshop Ver 23.1.1 to create panorama images and exported as full‐sized tiff files for analysis.

## 3 RESULTS

### 3.1 Histological results

**FIGURE 2:**
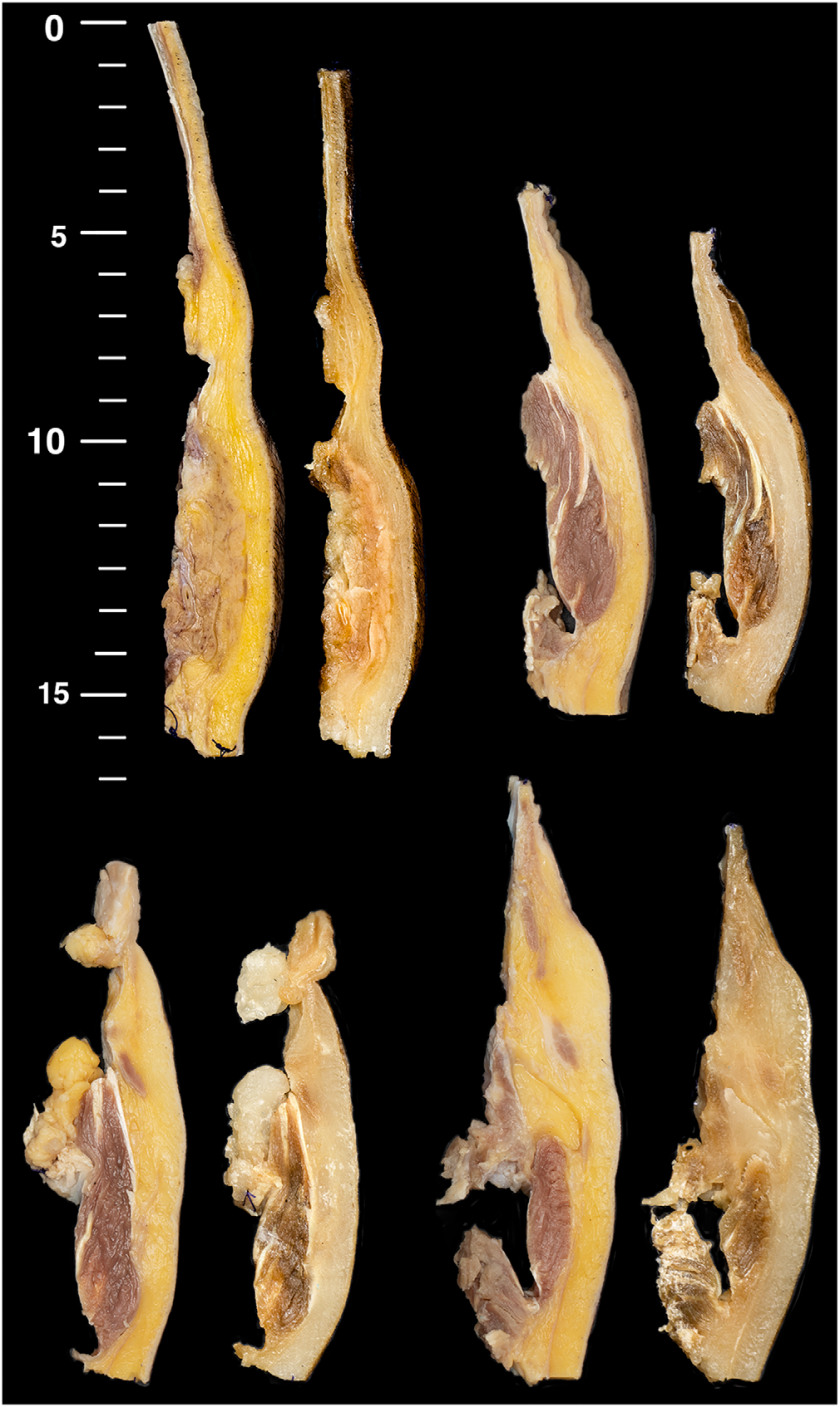
Shrinkage of tissues due to processing is demonstrated by comparing samples before and after the 6‐day processing regime

Extended processing times resulted in shrinkage of the tissues (Figure 2). This was noticed after the first processing run and, for the remaining tissues, the length and width were measured before and after tissue processing to permit quantitation of tissue shrinkage. In the 22 tissues measured, the length decreased with a mean of 6.4% (range 3.6%–14%) and the width decreased with a mean of 11.6% (range 7.1%–16.7%).

The histological slides effectively revealed the different tissue types present within the samples. Masson's Trichrome stained the cell nuclei black, and the collagen fibers dark blue. Traditionally, Masson's Trichrome stains muscle fibers bright red, however, in these larger samples the muscles stained dark red to dark brown.

### 3.2 Anatomical results

Using the described technique, 73 histological slides were successfully produced for analysis. This histological technique clearly visualizes the structural architecture of the facial mimetic muscles, the muscles of mastication, and the investing connective tissues of the different fascial layers. Moreover, the myelinated nerve fibers of the facial nerve and trigeminal nerves stained well, using this technique, which allowed the study of their position relative to the facial soft tissue layers. Most importantly, the soft‐tissue architectural organization is effectively demonstrated, from the macroscopic structure to the fine microscopic level allowing the patterns and connections of retinacular fibers to be defined and used to determine areas of fixation and areas of gliding.

**FIGURE 3:**
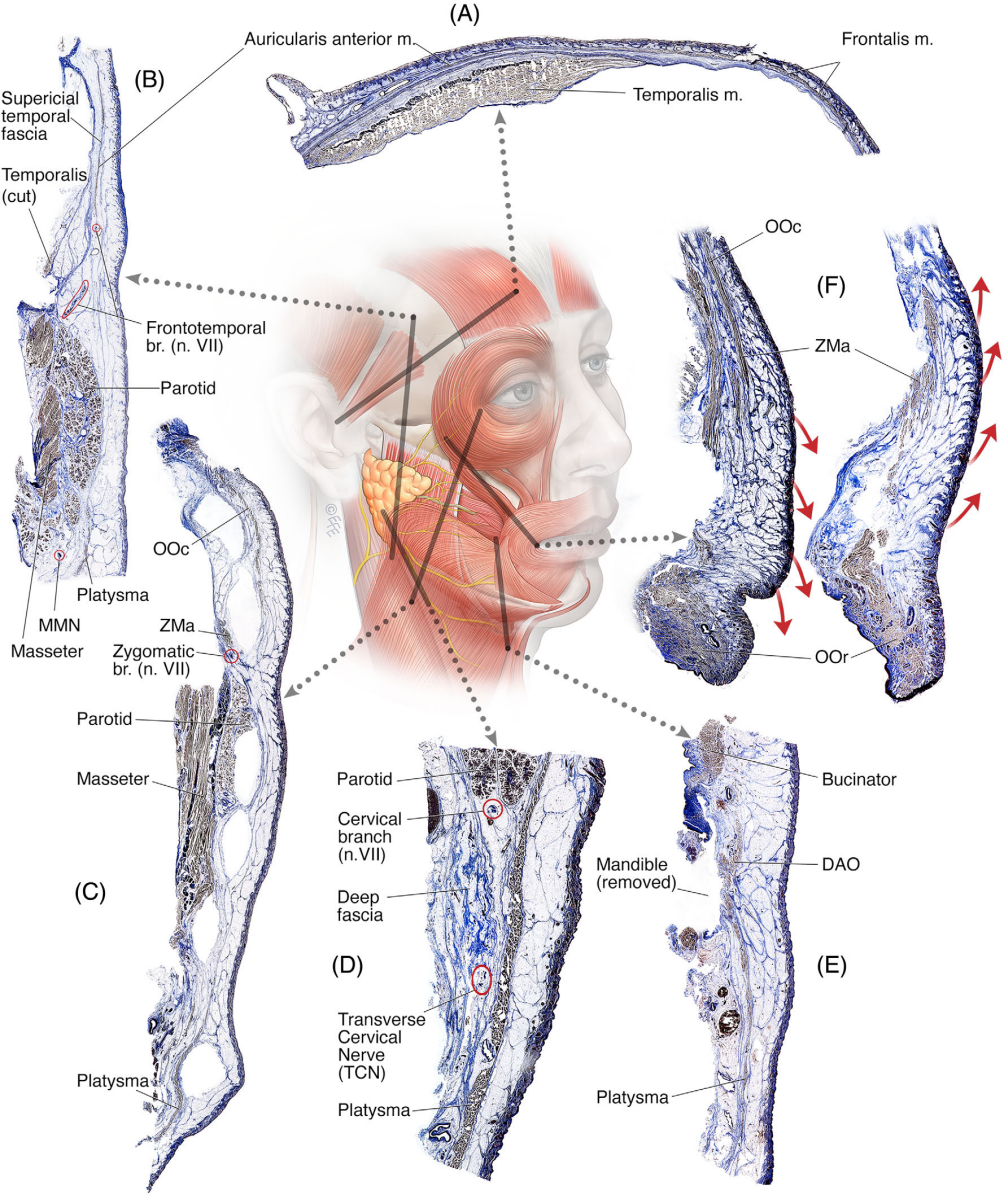
(A) 12 cm temple section of a 96‐year‐old male from the inferior upper tragal border to the forehead, demonstrating the auricularis anterior and frontalis muscles seen separating the subcutaneous layer from the deep fascia. (B) 13 cm lateral face sample of an 89‐year‐old female from the platysma to the superficial temporal fascia, demonstrating the SMAS in the lateral face connecting the platysma with the superficial temporal fascia (auricularis anterior muscle) over the parotid gland. The deep fascia is seen as a thick layer in the neck, to a very thin (to almost absent) layer over the parotid gland, to a thick layer over the zygomatic arch and then thin again over the temple. The frontotemporal branches are seen exiting the parotid gland at its superior border, traveling within the deep fascia to lie at the underside of the superficial temporal fascia in their trajectory to the forehead. (C) 17 cm sample of a 97‐year‐old female from the lateral canthus to the angle of the mandible demonstrating the surgical spaces created with a Trepsat scissors in the deep plane. Please note how the lower cervical space is erroneously created superficial to the platysma muscle: Shifting from deep to the platysma to within the platysma muscle or even superficial to the platysma muscle can occur in surgery when doing a blunt dissection blindly with the use of gentle spreading of a scissors. (D) 3 cm‐long neck sample of a 77‐year‐old male (BMI 25) at the anterior border of the sternocleidomastoid muscle (SCM). Note the continuation of the platysma over the parotid gland superiorly. The deep fascia is seen as a multilayered collection of fascial sheets. The cervical branch of the facial nerve is seen within the deep fascia (n. VII) as well as the transverse cervical nerves (TCN). (E) 7 cm‐long jowl sample of a 77‐year‐old female (BMI 26) through the maximal jowl fullness after removal of the mandible. The bony attachment of the platysma to the mandible is firm (*) and the overlying subcutaneous organization involves the collection of retinacula cutis in an obliquely hanging pattern opposing gravity, supporting the overlying dermis and providing length for lowering of the mandible when opening the mouth. (F) Two samples of the midcheek from the zygomatic body to the corner of the mouth on opposite sides of the same 69‐year‐old male (BMI 24). On the one side, a deep midcheek lift through the prezygomatic space was performed (B) and compared to the normal anatomy (A). Note how lifting of the cheek changes the direction of the retinacula cutis septa from a hanging‐down to a pulled‐up organization

Area‐specific results:

- *Temporal‐forehead sections* (Figure 3A) allow the study of the deep and superficial fascia in the temple and forehead and the relation with the frontotemporal branches of the facial nerve. The millefeuille‐pastry pattern of the deep fascia is well demonstrated as are the temporoparietal (auricularis) and frontal muscles.
- *Midcheek sections* (Figure 3B) allow the study of the fascial connections between the platysma muscle and the superficial temporal fascia, orbicularis oculi muscle, zygomaticus major muscle and upper lip levator muscles in the pursuit of the detailed description of the superficial musculoaponeurotic system (SMAS). Additionally, the depth of the facial nerve branches in their trajectory toward the anterior face can be studied.
- *Sections of surgically opened spaces* (Figure 3C) allow the study of the ligaments and soft‐tissue spaces of the face pertaining to natural gliding planes and facelift procedures.
- *Neck sections* (Figure 3D) allow the study of the connections of the platysma to the dermis superficially and to the deep fascia deep. Moreover, the relative depth of the marginal mandibular nerve branches and cervical nerve branches to the deep fascia and platysma are visualized allowing the study of the point of transition to a more superficial position, potentially dangerous during deep plane facelift procedures.
- *Jowl sections* (Figure 3E) allow the study of the mandibular ligament and architectural organization of the subcutaneous and deep soft tissues making up the bulging volume of the jowl.
- *Malar fat pad sections* (Figure 3F) on opposite sides of the same cadaver allow the comparison of a lifted cheek to a normal cheek using different techniques, to study the implications of facelift or thread‐lift procedures.

## 4 DISCUSSION

### 4.1 The study of facial surgical anatomy

Central in facelift procedures is the dissection of a wide area of the face and neck in one or more dissection planes (superficial/subcutaneous and/or deep/sub‐SMAS). An exact overview and study of the soft‐tissue architecture pertaining to these wide areas cannot be achieved by conventional small biopsy histology since surgically relevant structures are too large to be captured on a classic histology slide. Verification of connections between structures requires slides identifying all structures and the connections in between. The laudable work of Macchi et al. on the superficial musculoaponeurotic fascia, is a good example of limited small sections histology: the existence of a mimic connecting plate (MCP) between the platysma muscle and the zygomaticus major muscle was hypothesized without demonstrating this structure nor its connecting role between these two muscles (Macchi et al., 2009).

The early work of Barton et al. and Gosain et al. demonstrated that it is possible to conduct larger‐scale histology on facial soft tissues (Barton, 1992; Gosain et al., 1993). However, the absence of a reproducible histological technique has hindered the use of these techniques by less histology‐experienced researchers. Our histological methodology presented here, provides the clinical anatomist sufficient information to conduct structural and histomorphological investigation of facial architecture on large tissue samples from embalmed, fresh‐frozen or fresh cadavers. Upcoming publications by the authors will report on the detailed analysis of such large histological samples produced using this technique on the areas of the deep fascia, the superficial musculo‐aponeurotic system (SMAS), the spaces and ligaments, the malar fat pad, and the platysma. The detailed anatomy of the jowl area using this technique was recently published (Minelli et al., 2022).

Conventional computed tomography (CT) and magnetic resonance (MR) imaging, while able to study larger areas, lack the spatial resolution to perform microscopic differentiation. A new micro‐CT technique with improved spatial resolution has recently been introduced, providing promising results on facial soft tissue architecture (O et al., 2018). However, its maximal dimensions are limited by the scanner size of (limited to L 7 × W 7 cm) and adequate contrast agent penetration (limited to D 0.7 cm) (O et al., 2021). Additionally, imaging technologies cannot differentiate types of soft tissues (e.g., muscle cells from connective tissue cells), which would then be observer‐based and not entirely objective. For the purpose of conclusive anatomical research, where tracking anatomical structures over large distances is required, the histology technique here presented is superior to CT and MRI.

### 4.2 Optimizing histology technique

#### 4.2.1 Harvesting and preserving

Minimum sample thickness is limited by (1) manual sub‐sectioning which is prone to failure when cutting thinner than 7 mm and (2) required thickness for later microtomy sectioning along entire length of specimen allowing some alignment errors and tissue curling issues. Warping of samples is avoided by suturing the whole hemiface to rigid carboard prior to submersion in formalin, with sub‐sectioning into thinner samples 5 days later, after fixation has occurred.

#### 4.2.2 Processing

Early attempts at extended processing runs (21.5, 32.5, and 45‐h protocols) resulted in inadequate reagent infiltration, producing dry and distorted samples that were unsuitable for sectioning. The 12‐h step protocol (total processing time of 156 h) utilized in this research was adapted from the 1968 “Manual of Histological Staining Methods of the Armed Forces Institute of Pathology 3rd Ed” based on the ability to successfully prepare large specimens in the field (Luna, 1968). The combination of a pressure vacuum with each step, achievable with an automated tissue processor, assists with penetration of reagents into samples. In larger samples, inadequate reagent infiltration can result in cloudy paraffin embedding blocks with visible air bubbles producing flaky sections that do not remain intact while being collected or stained. Additional molten wax steps under pressure vacuum can correct these issues by gently forcing out trapped chemical residues, air, and water content.

#### 4.2.3 Sectioning

**FIGURE 4:**
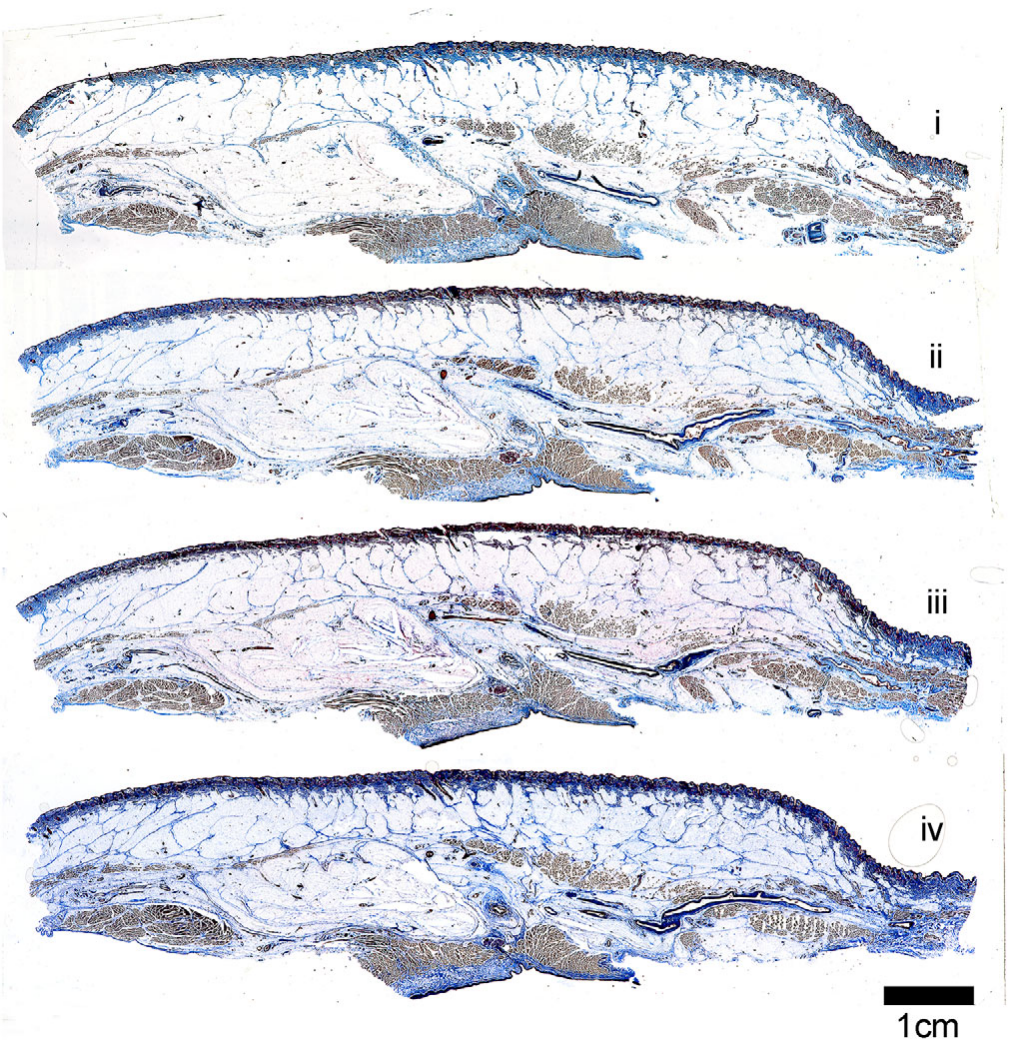
Differences in Masson's Trichrome staining in human cheek at varying section thickness. Four images of the same human cheek sample were initially compared to determine the optimum thickness for sectioning and staining of the soft tissue layer and muscle. Section thicknesses (top to bottom) were (i) 12, (ii) 20, (iii) 25, and (iv) 30 μm. The 30 μm section thickness was selected as the experimental section thickness due to the visualization of more intact retinacula cutis septa fibers compared to 12, 20, and 25 μm

Sections of 12, 20, 25, 30, and 40 μm thickness were collected for a staining trial (Figure 4). Individual cell resolution was slightly improved in sections of 12 μm than the thicker sections but the collagen‐rich retinacula cutis architecture of the superficial and deep fascial layers was not complete in these thinner sections. Sections of 20, 25, and 30 μm progressively demonstrated improved definition of the collagen‐rich architectural fibers. Sections at 40 μm produced uneven sections on the sliding microtome due to their excessive thickness. Sections of 30 μm provided the best overall results. C‐profile solid knives and disposable blades were trialed, with the latter being most suitable for this application.

#### 4.2.4 Collection

Uncoated glass slides, initially used for the collection and staining of sections, failed to retain intact sections due to lack of adhesion, often with complete section loss. Following this, gelatin‐chromium coated slides were trialed to collect sections floating on a warm water bath with no added gelatin. Although clearly improved, this similarly failed to retain intact sections. The unconventional combined use of gelatin‐chromium coated slides and a gelatinized water bath for section mounting was successful in ensuring section adhesion during the extensive staining process. Sporadically, minor histology artifacts still occurred, including tissue tearing from coverslip mounting. The gelatin‐chromium adhesive coating produced a consistent background staining artifact across all slides, which could be corrected with a correction of the white‐balance in the editing phase. It did not interfere with observation of anatomical structures of the sections.

#### 4.2.5 Staining

Masson's Trichrome stain was selected for its clear differentiation of collagen and muscle, necessary for demonstration of soft tissue architecture (Masson, 1929). Initial staining trials using an unmodified Masson's staining protocol produced understained muscle fibers and overstained collagen fibers. Subsequently, Biebrich‐scarlet red and aniline blue staining times were modified to optimize stain intensity of muscle and collagen, and acetic acid differentiation time was increased to improve the blue hue of the aniline blue. Some collagen fibers stained purple due to an overlapping false positive staining effect of Biebrich‐scarlet red. Phosphotungstic acid and phosphomolybdic acid differentiation times therefore were increased to ensure collagen fibers were adequately stripped of Biebrich‐scarlet red prior to aniline blue staining. The final protocol optimized for 30 μm sections of facial tissue is included in Table 2. Further optimization of staining for these tissues could allow for more traditional colorization of the staining technique in the place of the dark red and dark brown of the muscles.

#### 4.2.6 Embalmed versus frozen versus fresh cadaver tissues

Fresh‐frozen cadavers are generally more readily available to researchers than fresh non‐frozen cadavers. In the pursuit of making this technique available to the wider surgical community, the technique was tested on samples from cadavers that were previously fresh‐frozen (Figure 3A) or embalmed (Figure 3B). While these demonstrated equally good results, embalmed cadavers cannot be used to study the surgical manipulated architecture of soft tissue. The reason for this limitation is not because embalming prevents the surgical manipulation (soft embalming regimens allow surgical manipulation), but rather because previous embalming prevents subsequent fixation of surgically manipulated tissues in a fixed position.

### 4.3 Limitations

This study and technique have certain limitations. The technique is limited to samples that can fit the processing tray (L 17.5 × W 13.0 cm). This limitation cannot be overcome by manual processing, which would be too slow due to the absence of the vacuum and pressure steps. Our research has not been able to explain why the staining is different from traditional Masson trichrome stain and why it occurred more in some samples and less in other samples. It may be related to the thickness of our samples of 30 μm in contrast to traditional 12 μm thickness, and different fixation times for some of the samples, which was dependent on the transportation of samples between two labs with the samples being in formalin until arrival at the histology lab.

## INFORMED CONSENT

For this type of study informed consent is not required.

## Supporting information


**Appendix S1** Supporting Information.

**Video S1** Step‐by‐step demonstration of the tissue harvesting.

**Video S2** Step‐by‐step demonstration of the embedding.

**Video S3** Step‐by‐step demonstration of the sectioning.
