# Supplementary material for: Enhanced study of facial soft tissues using a novel large scale histology technique
Source: Clin Anat. 2022 Aug 17;36(1):110–7. doi: 10.1002/ca.23943 (PMC10086818; doi:10.1002/ca.23943)
Supplement: Supplementary file 1 — Appendix S1 Supporting Information. [file CA-36-110-s004.docx]

**Supplement 1.** Supplementary information for Masson’s Trichrome Staining solutions

Weigert's Iron Hematoxylin Solution:

Stock Solution A:

Hematoxylin ----------------------------- 1.25 g; Sigma Aldrich Pty Ltd: H9627

95% Ethanol ----------------------------- 125 ml;

Stock Solution B:

29% Ferric chloride in water --------- 5 ml; Sigma Aldrich Pty Ltd: 157740

Distilled water --------------------------- 120 ml

Hydrochloric acid, concentrated ---- 1.25ml; Thermo Fisher Scientific: R18603E

Weigert's Iron Hematoxylin Working Solution:

Mix equal parts of stock solution A and B. This working solution is stable for 3 months (no good after 4 months)

Biebrich Scarlet-Acid Fuchsin Solution:

Biebrich scarlet, 1% aqueous -------- 225 ml; Sigma Aldrich Pty Ltd:B6008

Acid fuchsin, 1% aqueous ------------ 25 ml; Sigma Aldrich Pty Ltd:F8129

Acetic acid, glacial ---------------------- 2.5 ml; Merck: 1000631011

Phosphomolybdic-Phosphotungstic Acid Solution:

Solution A

Phosphomolybdic acid ---------------- 6.25 ml; Sigma Aldrich Pty Ltd:221856

Distilled water --------------------------- 120 ml

Solution B

Phosphotungstic acid ------------------ 6.25 ml; Sigma Aldrich Pty Ltd:P4006

Distilled water --------------------------- 120 ml

Mix equal parts of stock solution A and B.

Aniline Blue Solution:

Aniline blue ------------------------------ 6.25 g; ChemSupply Aus: GT2666

Acetic acide, glacial -------------------- 5 ml; Merck: 1000631011

Distilled water --------------------------- 250 ml

1% Acetic Acid Solution:

Acetic acid, glacial ---------------------- 2.5 ml; Merck: 1000631011

Distilled water --------------------------- 250 ml
